# Precision Nutrition as the Concept of Dietary and Nutritional Interventions for Patients With Malignancies: A Narrative Literature Review and Perspectives

**DOI:** 10.1002/cnr2.70702

**Published:** 2026-10-06

**Authors:** Ken Yamaguchi, Koji Yamanoi, Jumpei Ogura, Ayami Koike, Maki Umemiya, Nozomi Higashiyama, Kazuhiro Kou, Yoshihide Inayama, Rin Mizuno, Taito Miyamoto, Mana Taki, Ryusuka Murakami, Junzo Hamanishi, Mandai Masaki

**Affiliations:** ^1^ Department of Gynecology and Obstetrics, Graduate School of Medicine and Faculty of Medicine Kyoto University Kyoto Japan; ^2^ Department of Obstetrics and Gynecology, Graduate School of Biomedical and Health Sciences Hiroshima University Hiroshima Japan

**Keywords:** cancer, chemotherapy, diet, food, molecular‐targeted therapy, nutrition, precision medicine

## Abstract

**Background:**

Dietary and nutritional interventions have gained popularity among patients with cancer undergoing treatment due to the high cost of medical treatment and increased health awareness. Diet, nutrition, psychological stress, and adverse events during treatment have a significant impact on the quality of life of patients with cancer. This study aimed to review published studies and guidelines on diet and exercise for cancer survivors.

**Recent Findings:**

Although several academic associations have published guidelines on the impact of diet and nutrition on cancer treatment, physicians typically do not consider this impact.

**Conclusion:**

This study summarizes recent findings on the effects of diet and nutrition, particularly fasting‐mimicking diet, on the prognosis and adverse events of chemotherapy. Furthermore, this study outlines future perspectives on cancer treatment based on “Precision Nutrition” and “Molecular Precision Medicine”.

## 1 Introduction

The use of diet and nutrition as therapies for patients with cancer has recently attracted much attention due to rising medical treatment costs and greater self‐care awareness during cancer treatment. Patients diagnosed with cancer often experience a decline in quality of life (QOL) due to the extreme emotional and physical effects they experience from diagnosis to posttreatment. Particularly, female patients with malignancies, including gynecological cancers, have a markedly lower QOL [1]. Diet plays a crucial role in a patient's QOL. However, chemotherapy‐related side effects, such as nausea, vomiting, anorexia, and infection caused by neutropenia, affect the diet and nutrition of patients.

Different diets, including the Atkins diet, caloric restriction, fasting, fasting‐mimicking diet, intermittent fasting, ketogenic diet, Mediterranean diet, time‐restricted feeding, vegan diet, and vegetarian diet [2], can be used to manage side effects such as anaplerotic reactions. Several organizations, including the American Cancer Society (ACS), American Society of Clinical Oncology (ASCO), American Institute for Cancer Research (AICR), National Cancer Institute (NCI), and European Society for Clinical Nutrition and Metabolism (ESPEN), have issued guidelines on diet and exercise for cancer survivors. Despite the availability of the guidelines, diet and nutrition support is not part of routine care [3].

In Japan, the Japanese Society for Parenteral and Enteral Nutrition (JSPEN) has published guidelines for venous enteral nutrition, including recommendations for patients with cancer. Unfortunately, many physicians overlook diet and nutrition management in their clinical experience due to insufficient evidence and awareness [3]. Dietary intervention is increasingly recognized as a feasible approach for patients with cancer. “Precision Nutrition” is an individualized nutritional strategy based on genomic information, metabolic status, microbiome composition, lifestyle behaviors, and environmental influences. The integration of “Precision Nutrition” with “Molecular Precision Medicine” may advance the delivery of truly precise medical care (Figure 1).

**FIGURE 1:**
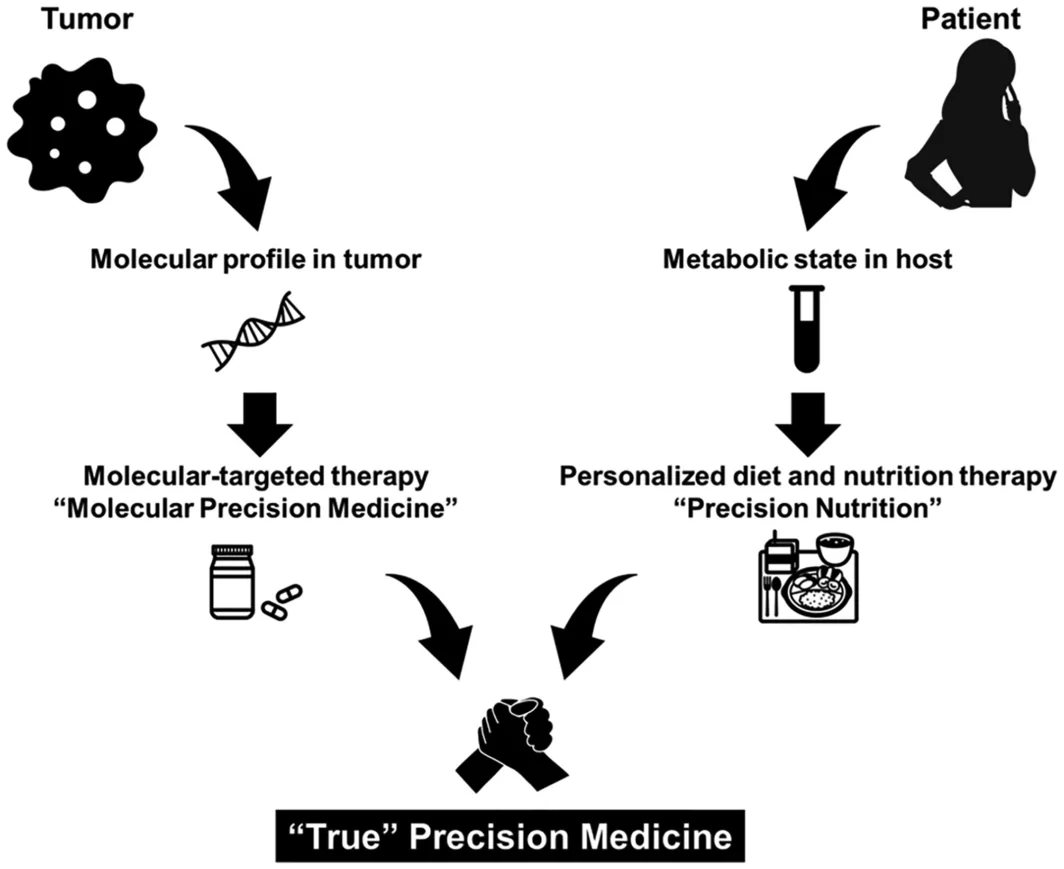
Proposal definition of true precision medicine. “True Precision Medicine” comprises personalized dietary and nutritional interventions (“Precision Nutrition”) and tumor profiling (“Molecular Precision Medicine”). *Source:* This figure was created by the authors and is not reproduced or adapted from previously published material.

Therefore, this review aimed to synthesize recent findings on the effects of diet and nutrition on the prognosis and adverse events of chemotherapy in patients with malignancies and to provide suggestions for future research in this field.

## 2 Materials and Methods

A literature search was conducted using the MEDLINE Public Library of Medicine database to identify relevant studies published between January 1, 2013, and July 31, 2023, using the following search terms: (diet OR nutrition) AND cancer AND chemotherapy. Randomized controlled trials, observational studies, feasibility or pilot studies, preclinical studies, editorials, and review articles describing the relationship between diet and chemotherapy were eligible for inclusion in this review.

## 3 Results and Discussion

### 3.1 Diet and Nutrition Mechanisms in Patients With Cancer

Diet exerts significant effects on the health and physiology of patients with cancer through several mechanisms, including modulation of cancer metabolism by nutrients, growth signaling, oxidative stress, gut microbiome and its derived metabolites, and immunity [2, 4]. Otto Warburg was the first to highlight the importance of metabolic reprogramming as a defining hallmark of cancer, which is now known as the Warburg effect [5], in which tumor cells use glucose to produce energy through aerobic glycolysis, regardless of oxygen availability and mitochondrial function, and convert glucose to lactate. This maximizes the ability of tumor cells to obtain energy and nutrients, making them vulnerable to changes in nutrient intake. Such effects can be used therapeutically.

Amino acids are associated with cancer development because cancer cells rely heavily on protein synthesis for growth and proliferation. Most cancer cells require an external supply of methionine for growth because they cannot synthesize it from homocysteine, which is known as the Hoffman effect [6]. Arginine is essential for tumor growth in high‐grade neuroendocrine carcinoma of the urinary bladder [7]. Furthermore, serine and glycine are involved in many processes that support tumor growth in intestinal cancer [8]. The glutamate transporter ASCT2 plays an important role in tumor growth in gastric, triple‐negative breast, and prostate cancers [9, 10, 11]. Several cancers rely heavily on specific amino acids obtained from the diet to sustain tumor growth. Lipids provide energy to tumor cells and play roles in metabolism and hormone production. The fatty acid receptor CD36 is associated with aggressive behavior in breast, ovarian, and oral squamous cell carcinomas [12, 13]. Dietary lipids such as palmitic acid and high‐fat diet can increase the metastatic potential of CD36^+^ metastasis‐initiating cells.

In addition to providing cellular nutrition, dietary interventions can impact tumor formation and development. Elevated glucose and amino acid levels in the bloodstream of patients with cancer can activate growth factor signaling by regulating p53, insulin, and insulin‐like growth factor 1, thereby promoting cell survival and proliferation through downstream signaling pathways such as PI3K/AKT/mTOR and Ras/MAPK [14, 15]. Adipose tissue aids in converting androgens to estrogen, which promotes tumor growth by enhancing signaling for proliferation and inducing angiogenesis, indicating a mechanism linking obesity with cancer [16].

Dietary interventions modulate oxidative stress signaling and gut microbiota. Hyperglycemia suppresses AMPK, thereby preventing the expression of the stress‐resistance factor early growth response protein 1 [17]. The gut microbiome, which is directly affected by diet, is essential for maintaining whole‐body energy homeostasis, intestinal development, mucosal barrier, and immunity. Therefore, gut microbiota dysbiosis is associated with numerous disorders, including metabolic syndrome and several malignancies [18, 19, 20, 21]. The gut microbiota plays a crucial role in tumor immunosurveillance, indicating that its modulation is effective in immunotherapy (Table 1) [31, 32, 33].

**TABLE 1:** Summary of human clinical and observational studies on dietary interventions in patients with cancer.

Dietary type	Cancer type/population	Study design	*n*	Intervention duration	Outcomes (summary)	Citation(s)
Fasting‐mimicking diet	Patients with early HER2‐negative breast cancer undergoing neoadjuvant chemotherapy	Multicenter randomized Phase II trial (DIRECT trial)	131 (65 fasting‐mimicking diet/66 control)	3 days per chemotherapy cycle (8 cycles = ~24 days)	Feasible and safe; ↓ grade III vomiting and neutropenia; ↓ IGF‐1 and hs‐CRP; biological signals of improved chemosensitivity.	[22]
Fasting‐mimicking diet + standard therapy (subanalysis)	Mixed solid tumors (subset of NCT03340935)	Subanalysis of a clinical trial	101 (analyzed)	Cyclic fasting‐mimicking diet during treatment (variable duration)	“Exceptional tumor responses” reported in a subset; exploratory evidence of synergic effect between fasting‐mimicking diet and anticancer therapies.	[23]
Fasting‐mimicking diet + vitamin C (adjunct therapy)	KRAS‐mutated solid tumors	Early phase clinical/observational adjunct study (pilot)	36	Short‐term cyclic fasting‐mimicking diet combined with vitamin C over multiple treatment cycles	Safe combination; metabolic reprogramming consistent with synergistic antitumor effects; signals of radiological responses in a subset.	[24]
Therapeutic/short‐term fasting around chemotherapy (systematic review and meta‐analysis)	Patients with mixed cancer undergoing chemotherapy	Systematic review + meta‐analysis of clinical studies	7 clinical studies (*n* = 300)	Fasting windows of 48–96 h around chemotherapy	Meta‐analysis: fasting or fasting‐mimicking diet reduced chemotherapy‐related fatigue, nausea, stomatitis, and hematologic toxicity; generally safe; evidence of moderate quality.	[25, 26]
Neutropenic/low‐bacterial diet	Patients with cytopenia after intensive chemotherapy for hematologic malignancy	Randomized controlled trial (Ann Oncol 2007)	80	During the cytopenic period (~14–21 days)	No reduction in infection rates; similar survival and complication rates compared with a normal hospital diet.	[27]
Neutropenic diet versus standard food safety	Pediatric oncology patients receiving chemotherapy	Pilot randomized clinical trial	27	During chemotherapy (~weeks)	Feasible; the incidence of infection was not significantly different between diets; similar nutritional outcomes.	[28]
Cooked versus noncooked diet	Acute myeloid leukemia during remission‐induction	Randomized clinical trial	150	During induction therapy (3–4 weeks)	No significant differences were observed in the infection rate or mortality; both diets were safe.	[29]
Low‐bacterial diet versus control	Patients with cancer with chemotherapy‐induced neutropenia	Cochrane systematic review (aggregate RCTs)	5 RCTs (*n* = 350)	During the neutropenic episodes	No clear benefit in infection prevention; current evidence is insufficient to recommend restrictive diet.	[30]
Dietary fiber/probiotic intake (observational study)	Patients with melanoma treated with anti‐PD‐1 immunotherapy	Prospective observational cohort study (Science 2021)	128	Baseline habitual diet assessed; follow‐up > 12 months	Higher dietary fiber intake was associated with better immunotherapy response and longer progression‐free survival; probiotic supplement use was linked to reduced diversity → poorer outcomes.	[31]
Microbiota signatures in the ICI response	Patients with melanoma treated with anti‐PD‐1 therapy	Observational study (Nature Med 2022)	222	Baseline → longitudinal (up to months)	Distinct gut microbiota compositions were correlated with clinical response and immune‐related adverse events; diet‐linked taxa were observed.	[32]
Fecal microbiota transplant (FMT)	Metastatic melanoma refractory to anti‐PD‐1 therapy	Phase II pilot clinical trial (Science 2021)	15	Single FMT + anti‐PD‐1 rechallenge; follow‐up ≥ 12 weeks	6/15 patients achieved renewed partial/complete responses; microbiome modulation could overcome resistance.	[33]
Dietary fat intake (CALGB 80405/SWOG 80405)	Metastatic colorectal cancer	Observational within the RCT cohort	1184	Habitual postdiagnosis diet assessment (follow‐up median≈15 months)	Higher total dietary fat was associated with ↑ all‐cause mortality; associations varied by fat type.	[34]
Red/processed meat intake	Stage III colon cancer (CALGB 89803)	Prospective observational	1011	Postdiagnosis dietary questionnaires; median follow‐up of 6.5 years	Higher red/processed meat intake → ↑ recurrence and mortality risk (adjusted HR≈1.7 for the highest versus lowest quartile).	[35]
Diet + lifestyle prediction model	Stage III colon cancer (CALGB 89803/Alliance)	Observational modeling study	1024	Longitudinal data over treatment and follow‐up	A validated model predicting recurrence/death from diet and lifestyle variables was developed, with dietary quality included in the predictive score.	[36]
Lifestyle adherence to the ACS/WCRF recommendations	Patients with high‐risk breast cancer	Longitudinal cohort	1340	Baseline → 2 years after treatment	Only≈15% fully adhered to all recommendations; higher adherence was associated with better weight control and QOL indices.	[37]
Food‐based interventions for chemoradiotherapy‐induced nausea/vomiting	Patients with mixed cancers undergoing chemoradiotherapy	Systematic review of clinical trials	9 RCTs (*n* = 560)	Peri‐treatment food‐based interventions (weeks)	Certain food‐based interventions (e.g., ginger, specific diets) reduced nausea/vomiting severity; moderate heterogeneity.	[38]
Nutrition status ↔ quality of life	Patients with ovarian cancer after surgery + chemotherapy	Observational study	202	During postoperative chemotherapy (≈6 months)	Better nutritional status was associated with higher QOL scores (EORTC‐QLQ‐C30); significant correlations were observed.	[39]
Nutrition literacy/self‐efficacy/social support ↔ dietary practice	Patient with breast cancer undergoing chemotherapy (multicenter, China)	Observational cross‐sectional	568	During chemotherapy	Higher nutrition literacy + self‐efficacy → better dietary practices; social support moderated this relationship.	[40]
Diet quality ↔ cognitive changes during chemotherapy	Patients with mixed cancer types undergoing chemotherapy	Prospective observational study (Support Care Cancer 2022)	80	Throughout chemotherapy (≈3–6 months)	Higher baseline diet quality was associated with a smaller decline in cognitive performance (*p* < 0.05).	[41]

Nutrition directly modulates immune function by providing the required energy and metabolic intermediates. Overnutrition contributes to tumor development through chronic inflammation‐associated interleukin 17 in adipocytes [42, 43]. Additionally, arginine metabolism in M1/M2 macrophages and selenium in T cells are important for antitumor immunity [44, 45]. Vitamins A and D induce the production of the anti‐inflammatory cytokine interleukin 10 by regulatory T cells, whereas vitamin C accumulation in T and B cells supports T‐cell maturation and B‐cell expansion, respectively [46, 47, 48].

### 3.2 Effect of Diet and Nutrition on Prognosis

The effects of a healthy lifestyle and diet on prognosis remain unclear. A previous study involving 1011 patients with Stage III colorectal cancer who consumed unprocessed and processed red meat for 6 months after chemotherapy showed no reduction in cancer recurrence or death risk among patients in the highest and lowest quartiles of red meat intake (Table 1) [35]. Furthermore, multivariable analysis revealed no reduction in the risk of recurrence or death, with hazard ratios (HRs) for recurrence or death of 0.84 (95% confidence interval [CI], 0.58–1.23) for unprocessed red meat and 1.05 (95% CI, 0.75–1.47) for processed meat.

However, several studies have shown that specific diets can improve the prognosis of patients with advanced and metastatic colorectal cancer. A previous study involving 1149 patients showed that higher fatty acids intake was associated with a lower risk of all‐cause mortality.

Similarly, patients in the highest quartile had a lower risk of cancer progression or death than those in the lowest quartile (Table 1) [34]. Furthermore, no other types of fat were associated with cancer progression or all‐cause or cancer mortality. Another study involving 1024 patients with Stage III colorectal cancer showed that incorporating the metrics of a healthy diet and lifestyle with clinicopathologic factors improved the accuracy of prognostic models for colorectal cancer (Table 1) [36]. Furthermore, the prognosis improved more in patients with higher clinicopathologic risk factors. A recent study showed that the risk of disease recurrence was 37.0% lower (HR, 0.63; 95% CI, 0.48–0.82) and the risk of mortality was 58.0% lower (HR, 0.42; 95% CI, 0.30–0.59) in patients with high‐risk breast cancer who adhered to lifestyle‐related cancer prevention guidelines (Table 1) [37]. Generally, patients with cancer who follow healthy lifestyle habits recommended in guidelines experience more favorable prognoses.

Several guidelines provide dietary recommendations for improving the prognosis of cancer survivors (Table 2). The ACS has proposed general diet and exercise recommendations for cancer survivors [49], citing many recommendations from the ACS Guidelines for Diet and Physical Activity for Cancer Prevention [50]. This guideline includes recommendations on dietary management during chemotherapy and describes the impact of diet and nutrition on cancer outcomes. The recommendations are as follows [49]:

- Nutritional assessments and counseling should begin immediately after diagnosis to prevent or resolve nutrient deficiencies, preserve muscle mass, and manage treatment side effects that may affect nutritional status.
- Physical and mental health assessments should begin immediately after diagnosis to help patients prepare for treatments, tolerate treatment, respond to treatments, and manage some cancer‐related symptoms and treatment‐related side effects.

**TABLE 2:** Comparison of the key nutritional guidelines in oncology.

Guideline/association	Target audience	Key recommendations for interventions and goals	Specific exclusions or cautions
American Cancer Society (ACS)	Cancer survivors; general recommendations	Begin nutritional assessments and counseling immediately after the diagnosis. Focus on preventing nutrient deficiencies, preserving muscle mass, and managing side effects. Maintain or increase muscle mass through diet and physical activity. Maintain a healthy eating pattern consistent with chronic disease prevention guidelines.	Avoid obesity
American Society of Clinical Oncology (ASCO)	Patients undergoing chemotherapy	Focus on exercise, diet, and weight management.	Do not recommend neutropenic diets for infection prevention in patients undergoing chemotherapy due to a lack of scientific evidence.
European Society for Clinical Nutrition and Metabolism (ESPEN)	Current patients and survivors	Current patients: Maintain a healthy diet and physical activity. If oral intake is inadequate despite counseling, consider supplemental enteral nutrition, followed by parenteral nutrition if enteral nutrition is unfeasible. Survivors: Maintain healthy weight (BMI 18.5–25 kg/m^2^); diet rich in vegetables, fruits, and whole grains; limit saturated fats, red meat, and alcohol consumption.	Benefits of glutamine supplementation during conventional chemotherapy or molecular‐targeted therapy remain unclear.
Japanese Society for Parenteral and Enteral Nutrition (JSPEN)	Current patients and high‐risk malnourished patients	Implement pretreatment nutritional assessments. Provide active intervention for malnourished patients or those at high risk of malnutrition. Intervene if oral intake is inadequate for approximately a week or if energy requirements are ≤ 60%. Prioritize oral nutritional supplements, followed by enteral and sparing intravenous nutrition. Target caloric goals: generally 25–30 kcal/kg/day.	No evidence indicates that nutritional interventions promote cancer growth. Intravenous nutrition should be administered sparingly.

Patients should be counseled to avoid obesity and to maintain or increase muscle mass through diet and physical activity appropriate to their type of cancer, health condition, treatment modalities, symptoms, and side effects to improve long‐term health and increase the likelihood of survival. Additionally, patients should maintain a healthy eating pattern that meets nutrient needs and aligns with recommendations of the ACS Guidelines for Diet and Physical Activity for Cancer Prevention to prevent chronic disease and reduce the risk of developing new cancers.

The AICR and the World Cancer Research Fund International (WCRFI) have published recommendations on diet, nutrition, and physical activity for specific malignancies (Table 2). In partnership with the AICR, the WCRFI developed the Continuous Update Project (CUP), which is a long‐term initiative that analyzes worldwide research to understand the impact of diet, nutrition, and physical activity on cancer risk and survival. The CUP report is an authoritative scientific resource that summarizes the strength of the evidence linking diet, nutrition, and physical activity with the risk of various types of cancer, as well as weight gain, overweight, and obesity [51]. Table 2 summarizes the nutritional guidelines used in oncology.

### 3.3 Effect of Diet and Nutrition on Adverse Events During Chemotherapy

#### 3.3.1 Low‐Bacteria and Neutropenic Diets

Diet management to mitigate the side effects of chemotherapy in patients undergoing chemotherapy is a way of improving QOL. Neutropenia, a common side effect of chemotherapy, is associated with an increased risk of life‐threatening infections. Low‐bacterial and neutropenic diets can help patients with cancer undergoing chemotherapy prevent such infections.

The search in the Cochrane Database of Systematic Reviews identified three randomized clinical trials involving 192 participants with different malignancies (Table 1) [27, 28, 29]. However, all three trials had methodological limitations that precluded pooling the data. Among these studies, two showed no significant differences in the infection rates between the intervention and control diet groups [28, 29]. Another study found no significant differences in the time from neutropenia onset to fever, duration of treatment with empirical antibiotics and antimycotics, diet acceptability, and QOL between the treatment arms (Table 1) [30]. Similarly, a meta‐analysis showed that low‐bacteria neutropenic diets had no preventive effect [52]. The ASCO based its recent recommendations on a review of a vast number of studies published between January 2000 and May 2021, including 42, 9, and 1 reviews on exercise, diet, and weight management, respectively, and 23 randomized controlled trials [53]. The ASCO guidelines do not recommend neutropenic diets for infection prevention in patients with cancer undergoing chemotherapy. However, evidence of the efficacy of other dietary and weight loss interventions during cancer treatment is limited [53]. Evidence demonstrating that a low‐bacterial diet can prevent neutropenic infections is lacking. However, during neutropenia, consuming fresh raw vegetables after washing with running water and eating cooked foods immediately after preparation is advisable.

#### 3.3.2 Management of Dietary Intake

Diet and nutrition can be used to manage other chemotherapy‐associated adverse events, such as nausea and vomiting. A systematic review of 17 trials showed that dietary counseling, peppermint drinks, scaly wood mushroom, chamomile, protein with ginger, and colorless and odorless diets significantly reduced nausea and vomiting in patients undergoing chemotherapy. However, evidence grades were generally low (Table 1) [38].

The NCI recommends nutritional therapy during cancer treatment to support cancer management and alleviation of side effects (Table 2) [52]. These guidelines are based on those provided by the ACS and AICR. The recommended behavioral strategies for symptom management are as follows [52]:

- **Loss of appetite and weight loss**. Eat small, frequent high‐protein and high‐calorie meals. Consume high‐protein foods first. Drink milkshakes, smoothies, juices, or soups. Look for foods that appeal to the sense of smell. Increase physical activity. Take good oral care and consider tube feeding if necessary.
- **Constipation**. Consume plenty of hot fluids every day. Eat more fiber‐rich foods. Increase physical activity. Limit the intake of certain foods such as broccoli, cabbage, and beans if the patient experiences gas. Consider taking a fiber supplement.
- **Diarrhea**. Drink plenty of fluids. Consume foods and liquids rich in sodium and potassium. Eat low‐fiber foods. Consume foods and beverages at room temperature. Avoid high‐fiber and high‐sugar foods, carbonated beverages, cruciferous vegetables, legumes, and chewing gum. Avoid milk products, alcohol, spicy foods, caffeinated drinks, and sugar‐free products sweetened with xylitol or sorbitol.
- **Nausea**. Consume bland, soft, and easily digestible foods. Eat dry foods, such as crackers, breadsticks, and toast throughout the day. Avoid strong food and bad smells. Avoid eating in a room with cooking odors or in a room that is overly warm. Sit or recline with your head raised for 1 h after eating. Rinse the mouth before and after eating. Consume hard candies such as lemon drops, if the mouth tastes bad. Eat small, frequent meals instead of three large ones. Do not keep your stomach empty. Consume liquids throughout the day and drink slowly. Sip small amounts of liquids during meals. Take meals that are neither too hot nor too cold.
- **Taste changes**. Use plastic utensils and do not drink directly from metal containers if the food or beverage has a metallic taste. Substitute red meat with poultry, fish, eggs, and cheese. Consume high‐protein foods other than meat. Add spices and sauces to foods. Consume marinated foods. Eat meat with something sweet. Try tart foods and drinks. Chew for longer periods to increase exposure to taste receptors if the sense of taste is dull. Avoid odors. Maintain good oral hygiene.
- **Dry mouth**. Sip water throughout the day. Consume sweet and tart beverages and foods such as gum and hard candy. Moisturize the food with sauces and salad dressings. Avoid alcohol, tobacco, and foods that may hurt the mouth. Keep your lips moist with lip balm. Rinse the mouth every 1–2 h.

The ESPEN has published practical guidelines on clinical nutrition in cancer [54] that cover general treatment concepts and specific interventions for different patient categories (Table 2), including current patients with cancer, cancer survivors, and those with untreated, advanced cancers. Patients with cancer should maintain a healthy diet and physical activity during chemotherapy. Supplemental enteral nutrition should be considered if the oral food intake is inadequate despite counseling and oral nutritional supplements. If supplemental enteral nutrition is not possible, parenteral nutrition may be necessary. Whether glutamine supplementation is beneficial during conventional chemotherapy or molecular‐targeted therapy is unclear. Cancer survivors should maintain a healthy weight lifestyle (body mass index [BMI] 18.5–25 kg/m^2^), which includes physical activity and a diet consisting of vegetables, fruits, and whole grains while limiting saturated fat, red meat, and alcohol consumption.

The JSPEN published the third edition of the Guidelines for Clinical Nutrition in Cancer in 2013 (Table 2) [55], which was updated in 2024. These guidelines recommend pretreatment nutritional assessments and active intervention should be implemented if the patient is malnourished or at high risk of malnutrition. During chemotherapy, patients' nutritional status should be assessed regularly with an active intervention provided if they are unable to maintain adequate oral intake for approximately a week. Intervention should be considered if the patient's energy requirements are ≤ 60%. Oral nutritional supplements should be prioritized, and enteral nutrition should be considered if oral supplements are not feasible. Intravenous nutrition is recommended if enteral nutrition is not possible due to the side effects of cancer treatment or if adequate nutritional intake cannot be achieved. Intravenous nutrition should be administered sparingly. Caloric goals should be calculated based on the patient's metabolic status and the invasiveness of the treatment. Generally, 25–30 kcal/kg/day should be considered, with adjustments over time based on patient‐specific factors. The JSPEN guidelines do not provide evidence of the impact of nutrition on treatment outcomes. No specific nutrients have been linked to cancer prognosis, and no evidence indicates that nutritional interventions during cancer treatment affect tumor growth. Table 2 shows a comparison of nutritional guidelines in oncology.

#### 3.3.3 Other Adverse Events

Doxorubicin damages cardiomyocytes and induces congestive heart failure. Intake of essential amino acids suppresses cardiotoxicity in mice via antioxidant effects [56]. Cancer and its treatment can lead to cognitive decline, affecting short‐term and working memory, attention, visuospatial skills, verbal fluency, and executive function. Consuming more monounsaturated and less saturated fats is recommended to reduce cognitive decline. This dietary change may help protect cognitive function and improve cognitive performance up to 5 days after chemotherapy infusion (Table 1) [41]. Monounsaturated fatty acids are healthy fats that have a positive effect on the body. However, consuming too many saturated fatty acids can negatively affect the body. Foods rich in monounsaturated fatty acids include olive, sunflower, canola, almonds, and macadamia nuts, whereas foods rich in saturated fatty acids include meat, beef tallow, lard, butter, dairy products, and palm oil.

### 3.4 The Fasting‐Mimicking‐Diet

Although many dietary and nutritional interventions exist, recent attention has been focused on fasting‐mimicking diet. Fasting‐mimicking diet was proposed in mouse xenograft models using breast cancer and malignant melanoma cell lines [57]. Fasting‐mimicking diet suppressed HO‐1 expression and decreased tumor growth by increasing tumor‐infiltrating lymphocytes [57]. A case series of patients with breast cancer receiving fasting‐mimicking diet for 5 days every 4 weeks and a mouse study showed that fasting‐mimicking diet prevented tamoxifen‐induced endometrial hyperplasia [58].

The DIetary REstriction as an Adjunct to Neoadjuvant ChemoTherapy for HER2 Negative Breast Cancer (DIRECT) trial phase II evaluated the effect of a plant‐based low‐amino acid diet for 4 days in patients with HER2‐negative breast cancer to determine whether fasting‐mimicking diet can serve as an adjunct to neoadjuvant chemotherapy. This trial demonstrated promising results, showing higher pathologic and imaging responses and less chemotherapy‐induced DNA damage in T lymphocytes in the fasting‐mimicking diet group (Table 1) [22]. The protocol for the DIRECT trial was as follows: on day 1, energy levels were limited to 1200 kcal and a carbohydrate: protein: fat ratio of 3.5: 1: 2; on days 2–4, carbohydrates were limited to 200 kcal and at least 80% of energy; and on day 5, patients consumed a recovery diet [22].

A 2022 Phase Ib clinical trial (NCT03340935) investigating the effect of fasting‐mimicking diet reported 5 cases that responded better than expected (Table 1) [23]. The clinical trial included several cases of cancer, including PD‐L1‐negative advanced small‐cell lung cancer, metastatic pancreatic adenocarcinoma, *KRAS*‐mutant metastatic colorectal cancer, and metastatic triple‐negative breast cancer. Patients were treated with fasting‐mimicking diet along with other treatments such as chemotherapy, bevacizumab, and anti‐PD1 antibody. The fasting‐mimicking diet protocol for this clinical trial included a reduced dose of 600 kcal/day with low protein and low‐carbohydrate foods on day 1 and 300 kcal/day on days 2–5. The treatment was administered every 3–4 weeks for up to 8 cycles [23]. Another study using a mouse *KRAS*‐mutant colorectal cancer cell line model showed that the effects of oxaliplatin were enhanced when combined with fasting‐mimicking diet and vitamin C (Table 1) [24].

Given that fasting‐mimicking diet restricts overall nutrition, potential adverse events and their impact on QOL remain concerns. In the DIRECT trial, the fasting‐mimicking diet and control groups showed declines in QOL scores, as measured by the European Organization for Research and Treatment of Cancer (EORTC) questionnaires (EORTC‐QLQ‐C30) and distress scores during treatment. However, the scores returned to baseline values 6 months after the surgery [21, 33]. Patients who received fasting‐mimicking diet had a better understanding of their treatment and were less concerned about its possible side effects than those on a regular diet [33]. Additionally, patients who received fasting‐mimicking diet demonstrated better emotional, physical, role, cognitive, and social functioning outcomes than those who did not receive fasting‐mimicking diet or were on a regular diet. Additionally, patients receiving fasting‐mimicking diet had lower symptom scores for fatigue, nausea, and insomnia [33]. A systematic review reported no difference in the incidence of grade ≥ 2 neutropenia and grade ≥ 2 adverse events in starvation therapy (Table 1) [25, 26]. However, few studies have been conducted, making it difficult to reach a conclusion.

### 3.5 Patients Eligible for Dietary and Nutritional Interventions

Providing dietary and nutritional interventions to all patients with cancer is not possible due to limited medical resources. Therefore, identifying the patients most in need of such interventions to improve their prognosis and QOL is important. A study conducted in China on 56 patients with ovarian cancer who were undergoing postoperative chemotherapy revealed that malnutrition levels, as determined by hypoalbuminemia or C‐reactive protein (CRP), remained consistent throughout the cycles of postoperative chemotherapy. In the first cycle, 7 patients (12.5%) were malnourished, whereas in the second cycle, 9 patients (18.8%) were malnourished. In the third cycle, the proportion of patients with malnutrition remained at 7 (15.5%) (Table 1) [39]. The study showed that only cognitive function from EORTC‐QLQ‐C30 affected BMI, whereas CRP significantly affected global health during the third cycle of chemotherapy. Therefore, in addition to directly measuring BMI, clinical parameters such as serum CRP levels should be considered [39].

Another study in China, involving 293 patients with breast cancer undergoing chemotherapy, was conducted to investigate factors influencing dietary practices (Table 1) [40]. Nutrition literacy, self‐care, self‐efficacy, and perceived social support were positively associated with dietary practice. Furthermore, nutrition literacy, self‐care, self‐efficacy, perceived social support, living environment, cancer stage, BMI, chemotherapy cycle, and average monthly household income were the main factors influencing patients' dietary practice (*p* < 0.05). The finding indicates that dietary interventions should be prioritized for patients who are obese, have a high income, live in rural areas, have low educational levels, have Stage I cancer, and receive multiple cycles of chemotherapy. Table 1 summarized the outcomes of human clinical and observational studies on dietary interventions in patients with cancer.

### 3.6 Future Directions

#### 3.6.1 Current Clinical Trials of Dietary and Nutritional Interventions

Table 3 summarized the current clinical trials of dietary and therapeutic interventions [2]. The use of dietary interventions to manage cancer, mainly through nutrient‐restricted approaches such as the fasting‐mimicking diet, caloric restriction, ketogenic diet, and amino acid‐restricted diets, has attracted considerable attention. These interventions are used in the treatment of many types of breast cancer. Conventional cytotoxic chemotherapy is used in most therapeutic interventions. Additionally, researchers have attempted to use PI3K inhibitors because the PI3K pathway is involved in the mTOR pathway, which affects glucose and lipid metabolism. Therefore, combining this approach with dietary and nutritional interventions holds considerable promise from a molecular biological perspective.

**TABLE 3:** Current clinical trials of dietary intervention combined with pharmacologic intervention.

Dietary intervention	Pharmacologic intervention	Type of cancer	NCT number
Fasting	Chemotherapy	Advanced and metastatic malignant skin neoplasms	NCT04387084
	Chemotherapy	Breast cancer	NCT05327608
	Chemotherapy	Breast cancer	NCT05259410
Fasting‐mimicking diet	Chemotherapy	Breast cancer	NCT04248998
	Chemotherapy	Breast cancer (HR+ and HER2−)	NCT05503108
	Chemotherapy	Lung adenocarcinoma	NCT03709147
Calorie restriction	Chemotherapy	Non‐Hodgkin's lymphoma	NCT05376709
	Chemotherapy	Breast cancer	NCT03131024
	Chemotherapy	Breast and prostate cancers	NCT01802346
	Chemotherapy	Hematological cancers	NCT05082519
Ketogenic diet	Chemotherapy	Breast cancer	NCT05234502
	Chemotherapy	Pediatric brain tumor	NCT03591861
	PI3K inhibitors	Follicular lymphoma and endometrial cancer	NCT04750941
	Chemotherapy	Pancreatic ductal adenocarcinoma	NCT04631445
	PI3K inhibitors	Breast cancer	NCT05090358
	Radiotherapy and chemotherapy	Glioblastoma	NCT03451799
	PI3K inhibitors	Glioblastoma	NCT05183204
Amino acid restriction	Chemotherapy	Metastatic pancreatic cancer	NCT05078775
	Chemotherapy	Metastatic colorectal cancer	NCT05183295

#### 3.6.2 Future Clinical Trials

Current clinical trials mainly use dietary and nutritional interventions in combination with cytotoxic agents. Therefore, further studies are urgently needed to investigate therapies that combine diet and nutrition with molecular‐targeted therapies, including PI3K inhibitors (Table 4) [2, 59]. These biomarkers are associated with cancer‐related gene pathways that affect several metabolic processes.

**TABLE 4:** Dietary and nutritional interventions plus pharmacologic therapies and future directions.

Type of cancer	Tumor subtype or biomarkers	Notable growth substrates	Potential dietary interventions	Potential pharmacologic therapies
Breast cancer	*PIK3CA* mutation	Glucose	Very low‐carbohydrate diet or fasting‐mimicking diet	PI3K‐mTOR pathway or an ER inhibitor
	Triple‐negative	Glucose or fatty acids	Low‐fat diet or fasting‐mimicking diet	CDK4/6 or an EGFR inhibitor
	PHGDH elevation	Serine	Serine‐ and glycine‐depleted diet	PHGDH inhibitor
Endometrial cancer	PI3K–mTOR pathway activation	Glucose	Very low‐carbohydrate diet	PI3K–mTOR pathway inhibitor
Prostate cancer	MYC pathway activation	Fructose or fatty acids	Low‐fat diet, calorie restriction or fasting‐mimicking diet	DNL pathway inhibitor
Lung cancer	*KRAS* mutation	Branched‐chain amino acids, ketones, fructose, or lactate	Unknown	Immunotherapy or chemotherapy
Pancreatic cancer	*KRAS* mutation	Fatty acids	Calorie restriction or a very low‐carbohydrate diet with high saturated fatty acids	SCD inhibitor or chemotherapy
Intestinal cancer	Wnt pathway activation	Glucose or fatty acids	Low‐fat diet	DNL inhibitors or chemotherapy
	SLC2A5 or KHK elevation	Glucose or fructose	Low‐sugar diet	KHK inhibitors, DNL inhibitors, or chemotherapy
Liver cancer	Microbiome alterations	Fructose, fatty acids, or short‐chain fatty acids	Low‐fat diet	DNL pathway inhibitor
Brain tumor	*IDH* mutation	Glutamine, serine or acetate	Very low‐carbohydrate diet or serine‐ and glycine‐depleted diet	PI3K‐mTOR pathway or PHGDH inhibitor

Evidence from preclinical studies can guide the development of future clinical trials of dietary and nutritional interventions. For example, PI3K‐mTOR inhibitors or ER inhibitors will be attempted with fasting‐mimicking diet and a very low‐carbohydrate diet in patients with breast cancer with *PIK3CA* mutations. This is because glucose is a significant growth substrate [58, 60, 61, 62, 63, 64]. Instead of targeting glucose metabolism or fatty acids, patients with triple‐negative breast cancer may benefit from combining CDK4/6 inhibitors or EGFR inhibitors with a low‐fat or fasting‐mimicking diet [65, 66, 67]. A serine‐ and glycine‐depleted diet along with a D‐3‐phosphoglycerate dehydrogenase (PHGDH) inhibitor may be beneficial for patients with breast cancer with elevated PHGDH [68]. Among gynecological cancers, uterine endometrial cancer has been reported to activate the PI3K‐mTOR pathway. Therefore, the combination of a very low‐carbohydrate diet and a PI3K‐mTOR pathway inhibitor targeting glucose metabolism is promising [60, 69, 70, 71]. The combination of a DNL pathway inhibitor and a low‐fat diet, calorie restricted diet, or fasting‐mimicking diet for patients with prostate cancer has been described [72, 73, 74, 75, 76, 77]. Other studies have reported the use of a combination of an SCD inhibitor and a calorie‐ or carbohydrate‐restricted diet with high saturated fatty acids for patients with pancreatic cancer [78, 79]. Furthermore, two previous studies reported a combination of a DNL inhibitor and low‐fat diets [80, 81] for patients with intestinal cancer. Additionally, a KHK or DHL inhibitor was previously combined with a low‐sugar diet to treat patients with gastrointestinal cancer [81, 82, 83]. Future studies should focus on DNL pathway inhibitors combined with low‐fat diets for patients with liver cancer [84, 85, 86, 87, 88, 89, 90] and on PI3K‐mTOR or PHGDH pathway inhibitors combined with very low‐carbohydrate or serine and glycine‐depleted diets for patients with brain tumors [91, 92, 93, 94, 95, 96].

Current precision medicine is based on the unique molecular profile of the tumor. However, because cancer progresses due to complex associations among genetic and environmental factors, individualized treatment is essential and should include the relationship between the patient's metabolic status and environmental factors (Figure 1). We propose the integration of “Precision Nutrition” and “Molecular Precision Medicine” as a conceptual framework termed “True Precision Medicine,” which warrants further investigation.

#### 3.6.3 Leveraging Digital Health Tools for “Precision Nutrition” Implementation

Incorporating digital technologies into medical care can facilitate the provision of more detailed, individualized, and data‐driven healthcare, thereby enhancing diagnostic accuracy, treatment planning, and patient outcomes [97]. The successful implementation of highly individualized nutritional strategies requires advanced methods for real‐time monitoring and support [98]. Digital health tools, including specialized mobile applications, wearable devices, and digital coaching platforms, offer a critical technological solution to support these complex dietary modifications in clinical practice. These tools can facilitate the real‐time collection of metabolic and dietary data, enabling practitioners to dynamically adjust personalized nutrition plans based on the patients' continuously changing physiological status (as is necessary for “Precision Nutrition”). Furthermore, digital platforms are vital for improving patient adherence to strict protocols, such as fasting‐mimicking diet or amino acid restrictions, by providing immediate feedback and support. By enabling scalable and precise monitoring, digital technologies will play a key role in accelerating the translation of nutritional interventions from controlled clinical trials to routine, individualized oncology care.

## 4 Conclusions

With the continuous rise in healthcare costs, the importance of dietary and nutritional interventions in cancer prevention, treatment, and avoiding cancer recurrence has increased. Although cancer treatment can impact a patient's diet by mitigating side effects, dietary and nutritional interventions can improve cancer treatment outcomes and reduce adverse events.

These findings can inform future clinical trials of combination therapies involving molecular‐targeted agents and chemotherapy supplemented with diet. “Precision Nutrition” encompasses personalized dietary and nutritional interventions tailored to each patient's metabolic status. Furthermore, “Molecular Precision Medicine” takes this personalization a step further by considering the molecular profile of the tumor. Collectively, we propose that the integration of these approaches may represent a future framework for “True Precision Medicine.”

## Author Contributions

**Ken Yamaguchi:** conceptualization, methodology, formal analysis, writing – original draft, visualization, project administration, investigation. **Koji Yamanoi:** investigation, writing – review and editing. **Jumpei Ogura:** investigation, writing – review and editing. **Ayami Koike:** investigation, writing – review and editing. **Maki Umemiya:** writing – review and editing. **Nozomi Higashiyama:** writing – review and editing. **Kazuhiro Kou:** writing – review and editing. **Yoshihide Inayama:** writing – review and editing. **Rin Mizuno:** writing – review and editing. **Taito Miyamoto:** writing – review and editing. **Mana Taki:** writing – review and editing. **Ryusuka Murakami:** writing – review and editing. **Junzo Hamanishi:** writing – review and editing. **Mandai Masaki:** writing – review and editing, supervision.

## Funding

The authors have nothing to report.

## Ethics Statement

The authors have nothing to report.

## Consent

The authors have nothing to report.

## Conflicts of Interest

Ken Y. and M.M. received a donation from DUMSCO Inc. Ken Y. and N.H. serve as advisors to DUMSCO Inc. and receive compensation for advisory services. This relationship may be perceived as a potential conflicts of interest. However, objectivity in the analysis and conclusions was maintained by adhering strictly to predefined methodological search criteria and ensuring a comprehensive critical review of all interpretations by the entire team of co‐authors.

## Data Availability

Data sharing not applicable to this article as no datasets were generated or analyzed during the current study.
